# Novel Differentially Methylated Regions Identified by Genome-Wide DNA Methylation Analyses Contribute to Racial Disparities in Childhood Obesity

**DOI:** 10.3390/genes14051098

**Published:** 2023-05-17

**Authors:** Priyadarshni Patel, Vaithinathan Selvaraju, Jeganathan Ramesh Babu, Xu Wang, Thangiah Geetha

**Affiliations:** 1 Department of Nutritional Sciences, Auburn University, Auburn, AL 36849, USA; 2 Boshell Metabolic Diseases and Diabetes Program, Auburn University, Auburn, AL 36849, USA; 3 Alabama Agricultural Experiment Station, Auburn University, Auburn, AL 36849, USA; 4 Department of Pathobiology, College of Veterinary Medicine, Auburn University, Auburn, AL 36849, USA; 5 HudsonAlpha Institute for Biotechnology, Huntsville, AL 35806, USA

**Keywords:** childhood obesity, epigenetics, health disparities, European American, African American, novel genes, DNA methylation

## Abstract

The magnitude of the childhood obesity epidemic and its effects on public health has accelerated the pursuit of practical preventative measures. Epigenetics is one subject that holds a lot of promise, despite being relatively new. The study of potentially heritable variations in gene expression that do not require modifications to the underlying DNA sequence is known as epigenetics. Here, we used Illumina MethylationEPIC BeadChip Array to identify differentially methylated regions in DNA isolated from saliva between normal weight (NW) and overweight/obese (OW/OB) children and between European American (EA) and African American (AA) children. A total of 3133 target IDs (associated with 2313 genes) were differentially methylated (*p* < 0.05) between NW and OW/OB children. In OW/OB children, 792 target IDs were hypermethylated and 2341 were hypomethylated compared to NW. Similarly, in the racial groups EA and AA, a total of 1239 target IDs corresponding to 739 genes were significantly differentially methylated in which 643 target IDs were hypermethylated and 596 were hypomethylated in the AA compared to EA participants. Along with this, the study identified novel genes that could contribute to the epigenetic regulation of childhood obesity.

## 1. Introduction

In 2016, approximately 340 million children and adolescents between the ages of 5 and 19 years, as well as 40 million children under the age of 5 years, were overweight or obese [1]. From childhood and throughout adulthood, this global epidemic of overweight and obesity poses substantial health risks. Overweight and obese children are a direct consequence of a long-term positive energy balance, which has its proximal determinants in the complex interactions among a person’s genetic makeup, lifestyle choices, an environment that promotes obesity, and social factors [2,3,4]. Even if the frequency of obesity among youth has stabilized, it has been significantly higher since the late 1980s for some population subgroups. During the period 1988–1994, only 8.6% of non-Hispanic White girls and 9.7% of White boys aged 2–19 years were obese, whereas obesity among non-Hispanic Black girls (14.5%) and Black boys (14.8%) was much higher. This trend is still continuing, with obesity prevalence being higher among non-Hispanic Black children (24.8%) compared to non-Hispanic White children (16.6%) in 2017–2020 [5]. These statistics signify the fact that obesity has disproportionately affected various ethnic groups, and it is important to understand the factors causing this disparity.

Despite studies showing that childhood obesity is heritable, the dramatic rise in the prevalence of obesity in children over the past few decades cannot be fully explained by changes in the genome due to the fact of evolution, suggesting that the childhood obesity epidemic is likely driven by gene–environment interactions [6,7]. Along with this expanding burden, obesity-related comorbidities, such as cardiovascular disease, diabetes, cancer, and musculoskeletal disorders, are becoming more common [8]. One of the main molecular mechanisms underlying the interaction between genes and the environment is epigenetics, which refers to mitotically inheritable changes in gene function that are not explained by modifications in the DNA sequence. It has been proposed that throughout development, epigenetics is highly susceptible to changes as a result of environmental factors and may set up metabolic and developmental pathways that lead to obesity [9,10]. DNA modification (DNA methylation and hydroxymethylation), noncoding RNA gene regulation, and chromatin and histone modifications are some of the main mechanisms involved in epigenetics. Although it is unclear if these processes are hierarchically arranged, they interact dynamically, with changes at one epigenetic layer reflecting those at others. Additionally, the genesis of childhood obesity may be influenced by epigenetics, but metabolic changes brought on by obesity may also have an impact on epigenetic modifications [11,12]. Epigenetic occurrences are also thought to play a role in an individual’s susceptibility to developing diseases, according to growing data.

Obesity is not just the result of excessive food intake or an imbalanced energy level. Instead, a wide range of epigenetic processes are thought to contribute to diet-induced obesity. According to Maugeri and Barchitta [13], association studies have linked particular eating habits with DNA methylation profiles in people. Preclinical studies showed that high-fat, high-calorie diets alter the methylation of genes that control metabolism and hunger, including the adipose tissue’s leptin (LEP) and the brain’s melanocortin 4 receptor (MC4R) [14]. Along with this, previous research shows that dietary lipids and carbohydrates produce changes at different CpGs that influence how genes are regulated, which in turn affects numerous metabolic pathways, increasing the likelihood of obesity [15].

Furthermore, several epigenome-wide association studies (EWAS) have shown that the characteristics of obesity are linked to changes in DNA methylation in various tissues [16,17,18]. DNA methylation (DNAm), which is the covalent addition of a methyl group to DNA and most typically occurs at cytosine–guanine dinucleotides, is the most studied epigenetic mark in humans (CpGs) [19]. Both the capabilities of technology for measuring DNA methylation and the range of useful bioinformatics techniques are advancing quickly. The Illumina Infinium Human MethylationEPIC (EPIC array), which targets nearly twice as many CpG sites, has recently superseded the most widely used DNAm microarray, the Illumina Infinium Human Methylation450 (450 K array) [20]. The Illumina Infinium MethylationEPIC array enables the quantification of over 860,000 CpG sites and increases coverage of genomic regions, including enhancers and noncoding regions due to the fact of additional content. The EPIC array has more than 94% of the 450 K material and often employs the same DNAm measuring methodology [21]. The main aim of this research was to identify the differentially methylated regions (DMRs) between normal weight (NW) and overweight (OW)/obese (OB) children and between European American (EA) and African American (AA) children using the Illumina Infinium MethylationEPIC array. We also determined the role of race, maternal education, and family income by multinominal hierarchical regression analysis. Along with this, we determined the genes and target IDs that overlap between BMI and racial groups. These differentially methylated regions in particular genes can also impact other biological processes and be identified through pathway analyses.

## 2. Materials and Methods

### 2.1. Participants

From Lee County and Macon County in Alabama, a total of 31 study participants between the ages of 6 and 10 years were recruited. After a preliminary phone interview with the parents, children with a history of cardiovascular illness or diabetes were excluded. The participant’s and their parent’s written consent were acquired. For the collection of saliva samples, the parents brought their children to Auburn University. The study received approval from Auburn University’s Institutional Review Board.

### 2.2. Anthropometric Measurements

The WHO’s recommendations were followed for taking the participants’ anthropometric measures. The children’s body weight was measured on a Tanita digital scale to the nearest 4 ounces. Without shoes and in light clothing, their weight was assessed. Height was measured with a calibrated scale that was attached to a stadiometer, and the accuracy was 0.1 cm. Boys and girls have different ratios of bone, muscle, and fat as they get older and, as a result, the BMI z-score (body mass index) was calculated using the SPSS macro, which is based on the WHO’s growth references, adjusted for age and sex. The BMI z-score is a better statistic for analyzing a developing child’s weight than only BMI. According to the guidelines of the Centers for Disease Control and Prevention (CDC), children were categorized as underweight (<5th percentile), normal weight (>fifth percentile to the <85th percentile), overweight (>85th percentile to the <95th percentile), and obese (>95th percentile). Based on these guidelines, the participants were categorized as normal weight (NW), overweight (OW), or obese (OB).

### 2.3. Isolation of Salivary DNA

The Oragene Geno-Tek saliva collection kit (Catalog #OGR-500; Ottawa, ON, Canada) was used to collect saliva. The saliva samples were incubated in a water bath at 50 °C for three hours, as directed by the manufacturer. The PrepIT.L2P DNA isolation kit (Catalog #PT-L2P-5; DNAgenotek, Ottawa, ON, Canada) was used to isolate DNA from a 500 µL aliquot. Each sample was labeled and kept at −20 °C. The quantity of isolated DNA was measured using a NanoDrop ND-1000 spectrophotometer (Thermo Fisher Scientific, Inc., Wilmington, DE, USA).

For further evaluation, the samples were sent to the University of Minnesota Genomics Centre for the Illumina Infinium MethylationEPIC BeadChip method analyses.

### 2.4. Sodium Bisulfite Conversion and Infinium Arrays

Using the EZ DNA methylation kit, sodium bisulfite was used to treat DNA (250–750 ng) (Zymo Research, Irvine, CA, USA). The manufacturer’s recommended methodology was followed for measuring DNA methylation using the Illumina Human MethylationEPIC BeadChip (Illumina, CA, USA). The bisulfite-treated samples were amplified, fragmented, purified, and hybridized onto the EPIC BeadChip. Using the Illumina HiScan System, the arrays were cleaned and scanned.

With Illumina’s GenomeStudio software V2011.1, raw IDAT data were processed, and background normalization using negative control probes produced methylation β-values that were used for all subsequent analyses. For processing the EPIC data, we used MethylationEPIC v-1-0 B2 manifest.

### 2.5. Data Analysis

From GenomeStudio by Illumina, signal intensities and raw methylation levels were extracted. Probes that had data from just two beads or fewer or those that had signal detection *p*-values higher than 0.01 were eliminated. To obtain methylation β levels, the signal intensities were standardized, and the noise was eliminated using negative control probes. The methylation β values were derived as the ratio of methylation probe intensity to the overall intensity. IBM SPSS version 24.0 and the R macro-package were used for the statistical analysis. From the 862,927 CpG sites analyzed, a total of 823,645 target IDs were used for further analysis after a quality check. We used a *t*-test analysis, as shown in Table 1, to measure the difference between weight and category of race. Multinomial hierarchical regression analysis was performed to measure the relationship between the BMI z-score and target IDs, adjusting for covariates (Table S4). To obtain the differences in the methylation status of significant target IDs between NW and OW/OB subjects, an independent sample *t*-test was conducted. A similar calculation was performed to determine the target IDs that were significantly different between EA and AA subjects. To account for the false discovery rate, a Q-value for each sample was calculated, where a Q-value of 0.05 indicates that 5% of significant tests will result in false positives. Differentially Methylated Regions (DMR) were identified based on the Q-value. The percent difference was calculated by subtracting the average β methylation value of the target ID of OW/OB children and AA children from the average methylation of NW and EA children, respectively, and multiplying it by a hundred. Furthermore, using the list provided by the DisGeNET (https://www.disgenet.org, accessed on 12 August 2022) platform, one of the largest databases for genes and genomes, we chose the target IDs of the 2822 obesity-related genes for further analysis.

## 3. Results

### 3.1. Study Participants

For the Infinium MethylationEpic BeadChip Array, we used DNA samples from 31 children: 11 NW and 20 OW/OB. The participants were also divided into different races: 16 EA and 15 AA. Table 1 shows the general characteristics of the study population. The mean age and height were not statistically significant among both groups and, as expected, OW/OB children had higher anthropometric measurements compared to NW children. The body weight (kg) of OW/OB (40.33 ± 12.72) was statistically (*p* < 0.001) higher compared to NW (27.16 ± 1.89) children. Similarly, the BMI (kg/m^2^) of OW/OB (2.03 ± 0.10) was significantly greater than that of NW (0.09 ± 0.31). However, between the racial groups (i.e., EA and AA), we did not see any statistical differences in the anthropometric measurements.

### 3.2. Identification of Differentially Methylation Regions

**Figure 1:**
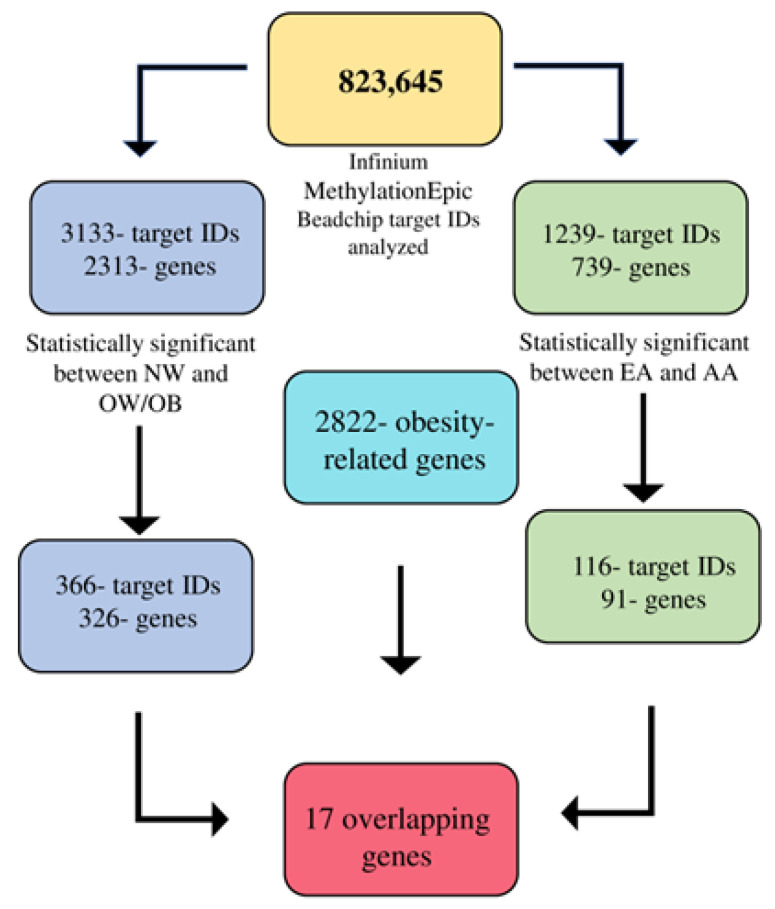
Workflow of Infinium MethylationEpic BeadChip target IDs analyses.

To demonstrate the DMR between NW and OW/OB children and between EA and AA children, we conducted an independent sample *t*-test between the groups. Figure 1 shows the workflow of the differentially methylated regions (based on the Q-value). A total of 3133 target IDs (associated with 2313 genes) were differentially methylated (*p* < 0.05) between NW and OW/OB children (Table S1). Among them, 792 target IDs were hypermethylated, and 2341 were hypomethylated in OW/OB children compared to NW participants. From those target IDs, we identified 366 target IDs corresponding to 326 unique genes that are associated with obesity that were differentially methylated between NW and OW/OB children (Table S2). The target IDs that are non obesity related and are differentially methylated between NW and OW/OB children are listed separately in Table S3. To determine the role of maternal education and family income on the significant target IDs between NW and OW/OB children, we conducted a multinominal hierarchical regression analysis between the obesity-related target IDs and the BMI *z*-score of the children. Out of the 366 significant obesity-related target IDs, 40 target IDs were not significant after adjusting for maternal education, family income, and race (Table S4). Similarly, for the racial groups EA and AA, a total of 1239 target IDs corresponding to 739 genes (Table S5) were significantly differentially methylated of which 643 target IDs were hypermethylated and 596 were hypomethylated in AA in comparison to EA participants. A total of 116 target IDs were obesity-related corresponding to 91 genes (Table S6), and 1123 target IDs were nonobesity related (Table S7).

### 3.3. Methylation Distribution and Classification Analysis

**Figure 2:**
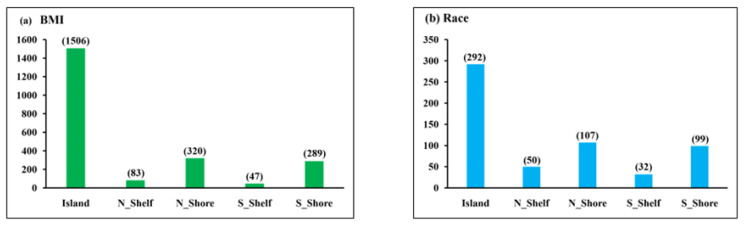
Genomic location of significant target IDs between (**a**) BMI and (**b**) race. Green color is for BMI group and blue for racial group.

Methylated cytosines can be found in gene bodies, 3’ untranslated regions (UTRs), other/open sea areas derived from genome-wide association studies, CpG islands, shores, shelves, open seas, and sites surrounding the transcription sites (200 to 1500 bp, 5’ UTR, and exons 1) for coding genes. The regions 0–2 kb from CpG islands is referred to as shores, the regions 2–4 kb from CpG islands are referred to as shelves, and the other/open sea regions are isolated CpG sites in the genome that do not have a specific name [22]. Figure 2 shows the distribution of the genomic location of the significant differentially methylated regions between NW and OW/OB children and between EA and AA children, where most of the target IDs in both groups were islands, 1506 and 292 for BMI and racial group, respectively. Table 2 and Table 3 show some clusters of target IDs of the top obesity-related significant genes with a methylation difference of more than 10%. Some of the top hits for genes differentially methylated between NW and OW/OB children were *PCDHGA4*, *BRD2*, *PTPRN2*, and *DIP2C*, while those between the racial groups EA and AA were *PTPRN2*, *TNXB*, *MDLL1*, and *PRDMI6*.

**Figure 3:**
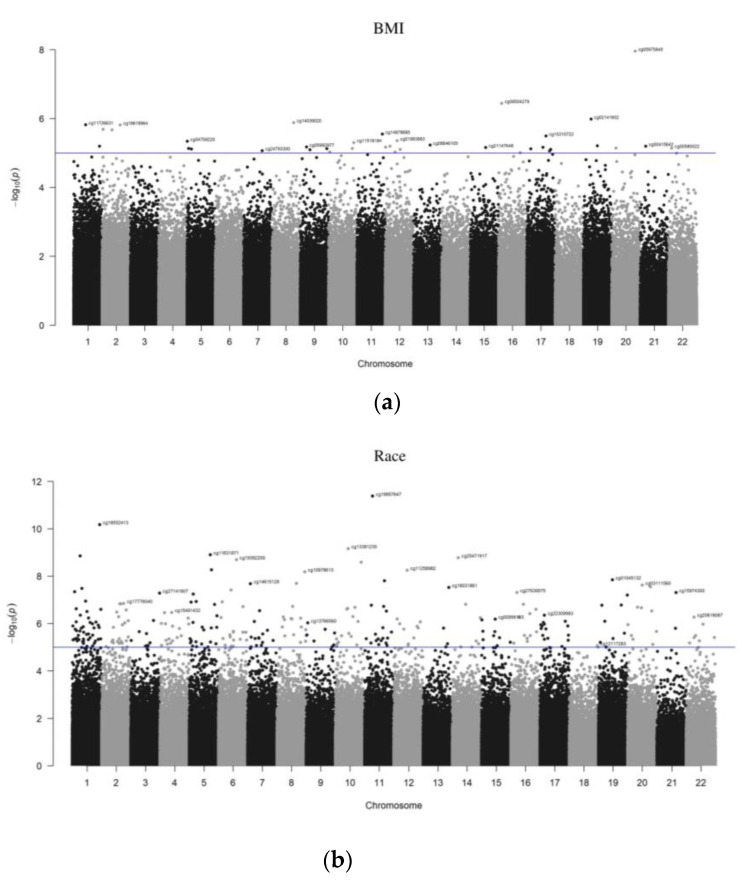
Manhattan plot of DMR between (**a**) NW and OW/OB (**b**) EA and AA children. This figure illustrates the position of each CpG arranged by chromosomal location (xaxis) and the level of statistical significance measured by the negative logarithm of the corresponding *p*-value (*y*-axis). The blue line corresponds to the threshold of *p* = 1.00 × 10^−5^.

**Figure 4:**
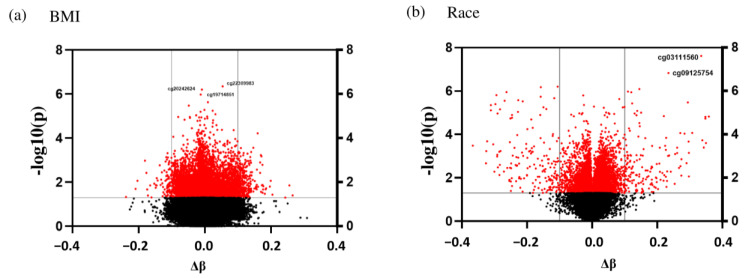
Volcano plot of the DMRs between (**a**) NW and OW/OB children and (**b**) EA and AA children. The red dots are DMRs that were statistically significant at the *p* < 0.05 level, the black dots are non-significant target IDs. The dots between 0.0 and −0.4 are hypomethylated and between 0.0 and 0.4 hypermethylated. The vertical, gray lines represent the 10% cut-off for difference in methylation. The horizontal, grey lines correspond to a significance level of 0.05.

At the genomic scale, the Manhattan plots show the *p*-values for the complete epigenome-wide association studies (EWAS). The *p*-values for each target ID are shown in Figure 3a,b by chromosome and position on the chromosome in genomic order (*x*-axis). The number on the *y*-axis represents the *p*-values log10 value, which is equal to one more zero after the decimal point. The Manhattan plots were created using the qqman software in the R-package to illustrate the chromosomal distribution of the significant associations, and volcano plots are shown in Figure 4a,b to visualize the overall size and direction of the associations. From the volcano plots, some of the top hits for the BMI group included cg20242624, cg2230998, and cg19714851, while for the racial groups, the top target IDs were cg03111560 and cg09125754.

### 3.4. Pathway Analysis

The extended list of genes with *p* < 0.05 for NW vs. OW/OB were significantly enriched for Gene Ontology (GO) processes involved in carbohydrate kinase activity (*p* = 0.000059) and sequence-specific double-stranded DNA binding (*p* = 0.000388). Table 4 represents the genes involved in each of these processes. While for the genes that are significantly different between EA and AA, GO processes such as cholesterol binding (*p* = 0.009942), and sterol binding (*p* = 0.006447) were significantly enriched (Table 5). We also used the same list of genes to perform the Kyoto Encyclopedia of Genes and Genomes (KEGG) analysis. For the genes that were differentially methylated between the NW and OW/OB groups, pathways such as Hippo signaling pathway (*p* = 0.000429), folate biosynthesis (*p* = 0.001785), type II diabetes mellitus (*p* = 0.004951), and MAPK signaling (*p* = 0.008373) were enriched, as shown in Table 6. For the gene that was differentially methylated between EA and AA, pathways such as the Ras signaling pathway (*p* = 0.0126) and biosynthesis of unsaturated fatty acids (*p* = 0.016499) were enriched (Table 7).

**Figure 5:**
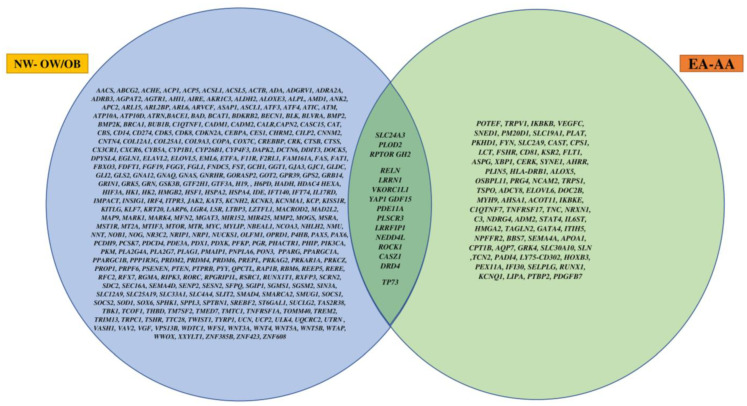
Venn diagram of overlapping significant genes between NW and OW/OB children and between EA and AA children.

Lastly, we found 17 obesity-related genes (Figure 5) that were differentially methylated between NW and OW/OB, as well as between EA and AA, children. Some of these genes were *SLC24A3, PLOD2, RPTOR, GH2, RELN, LRRN1, VKORC1L1,* and *YAP1*.

## 4. Discussion

In this study, we looked at the DMRs between NW and OW/OB children and also between EA and AA races. Overall, 17 obesity-related overlapping genes were found to be differentially methylated within both groups. Furthermore, the target IDs that were differentially methylated between NW and OW/OB children showed no effects on maternal education and family income.

Along with this, 366 CpG islands were identified for 326 genes that were related to obesity, among which the gene *PCDHGA4* (protocadherin γ subfamily A, 4) demonstrated the highest number of target IDs—52 target IDs that were differentially methylated between NW and OW/OB children. Previously, this gene has been associated with dysglycemia [23] but, according to our knowledge, this is the first study that shows its association with childhood obesity. The Brd2 (bromodomain containing 2) deficient model for “metabolically healthy” obesity has garnered attention recently and provides a novel interpretive tool. This model has an epigenetic basis for obesity. Targeted disruption of the Brd2 gene, which is present in the class II major histocompatibility complex (MHC) nearer to Tnf, results in extreme obesity with hyperinsulinemia, as well as hypoglycemia, hyperadiponectinemia, and improved glucose tolerance, which is quite different from other animal models of obesity [24,25]. *BRD2* had 18 target IDs that were hypomethylated in the OW/OB children compared to NW and based on previous literature, hypomethylation can cause a decrease in gene expression; therefore, the OW/OB children were hypomethylated. A significant islet autoantigen for type 1 diabetes is encoded by *PTPRN2* (protein tyrosine phosphatase, receptor type N2). Previous genetic research has demonstrated a strong correlation between it and obesity. With 12 hypomethylated and 3 hypermethylated sites in OW/OB children, this gene was one of the top hits in this study; unfortunately, the only two CpG islands that previously [26] showed an association between childhood obesity and BMI were not observed in this study, but the results showed novel sites that could play a role in gene expression. We also assessed the DMRs between the two racial groups EA and AA; the gene *PTPRN2* had 10 CpG sites hypomethylated in AA children compared to EA. Since for both groups in our study BMI and the racial groups showed clusters of CpGs hypomethylated in the later groups, the results provide us with insight into the potential role of *PTPRN2* hypomethylation linked with childhood obesity and racial disparities.

To understand the processes behind these associations, gene- and network-specific functional experiments are needed to determine the significance of pre-pregnancy obesity-related methylation levels identified in the *TAPBP* (TAP binding protein) gene. Recently, it was discovered that among five-year-old children, the hypermethylation of numerous CpG sites in *TAPBP* at birth was linked to an elevated risk of cardiometabolic outcomes (such as insulin sensitivity). Another study demonstrates that hypermethylation of *TAPBP* regulatory sequences may play a significant role in the association between maternal obesity and cardiometabolic dysfunction in children [27,28]. Our study showed 13 hypomethylated and 1 hypermethylated region in the OW/OB children compared to NW, implying further studies are needed to understand the epigenetic changes related to *TAPBP* and obesity. According to one study [29], DMR contains the gene *FDFT1*, which codes for the essential enzyme squalene synthase, which is involved in sterol biosynthesis. Individuals with abdominal obesity have been discovered to have higher visceral fat levels of *FDFT1* expression. It has been suggested to treat hyperlipidemia with *FDFT1* inhibitors. Furthermore, homozygous knockout mice have demonstrated that *FDFT1* is critical for liver function, central nervous system development, and cholesterol production. Children under the age of 10 who breastfed for more than three months have a large DMR across the gene *FDFT1*. Therefore, breastfeeding may, through methylation, have favorable effects on later life occurrences such as obesity, heart disease, and neurological function. In our study, *FDFT1* showed 11 DMRs that were hypomethylated in the OW/OB children. Further studies in obesity and breastfeeding through epigenetic changes in the *FDFT1* gene can provide new insight [30,31,32]. Looking at the DMR between EA and AA, *TNXB* was one of the top hits with nine different target IDs differentially methylated. It has been shown that with various child anthropometric parameters, EWAS have found hundreds of sites that were differentially methylated, with similarities between several studies for the four genes *HDAC4, PRLHR, TNXB*, and *PRDM16* [33,34,35,36,37]. Given that children’s obesity outcomes have been linked to the genes *PRLHR* and *TNXB*, these genes need further study.

Along with this, the top hits for the DMR between the NW and OW/OB children also included some novel genes that were not identified in previous studies to have been linked to childhood obesity and epigenetics. Those genes were *TGIF1, PITX2, COL9A3, PAX6, NKK6-2, SMC4, PCHDB16, TRIM27, BAHCC1*, and *NFIX*, and for the DMR between the EA and AA, genes such as *MDLL1*, *PRDMI6*, *CSMD1*, and *TRIM31* were among the top hits, but little to no information was found in the literature.

One of the findings of this study are the enrichment genes associated with various pathways and processes. Divers’ pathology and physiological processes, such as tissue repair, wound healing, tissue size, and tissue regeneration, are influenced by the Hippo signaling system, which has been one of the top pathways to be enriched in this study, as well as in previous research [38]. Histone acetylation and deacetylation, dysfunctional miRNAs, abnormal DNA methylation, and aberrant activation of inflammatory genes in the Hippo signaling pathway are all indications of epigenetic alterations [39]. An intervention study [40] observing the effect of a hypocaloric diet on the DNA methylation patterns showed that the MAPK signaling pathway was enriched and hypomethylated in women with a hypocaloric diet, the result of our study falls in line with this, where a cluster of genes with DMRs between NW and OW children were enriched for the MAPK pathway.

Emerging literature shows that the biosynthesis of unsaturated fatty acids is affected by the epigenome. The liver of mice fed a diet high in linolenic acid during pregnancy showed an increase in the DNA methylation status of the Fads2 promoter. In rats, increasing the amount of total maternal fat consumed during pregnancy and breastfeeding caused the Fads2 promoter to remain hypermethylated in the offspring’s liver and aorta. However, adult rats that consumed more fish oil experienced momentary, reversible hypermethylation of Fads2. The rodents on high-fat diets also had their placentas and adipose tissue’s histone methylation levels changed. Docosahexaenoic acid supplementation during pregnancy caused minor alterations in the overall DNA methylation of leukocytes from cord blood. A high-fat diet changed the DNA methylation state of several genes in young men’s skeletal muscle [41,42,43]. The genes that were differentially methylated between EA and AA showed enrichment in the biosynthesis of the unsaturated fatty acids pathway.

Our study has a few limitations as well, such as the relatively small number of participants. Nonetheless, we detected significant epigenetic differences. We did not consider the variations in the genome background of the subjects, which may explain a proportion of the epigenetic variation. Additionally, we want to establish that salivary samples can be used to determine DNA methylation. Previous research shows DNA methylation patterns, both at the gene-specific and global (hydroxyl) methylation levels, are comparable between blood and saliva. The genome-wide DNA methylation profiles of saliva in adults and adolescents are more than 90% similar to those in blood, according to a number of independent studies [44].

To conclude, we found novel genes that were differentially methylated between NW and OW/OB children and between EA and AA ethnicity, which further opens the window to explore the top hits and the overlapping genes in more detail. To our knowledge, this is the first study that explored the epigenomic differences in children who are NW and OW/OB, along with differences in the racial groups EA and AA. Our study also highlights the importance of the consideration of health disparities in epigenetics and obesity, as not many studies have been conducted.

## Figures and Tables

**Table 1:** General characteristics of the study population.

Parameter	Total	NW	OW/OB	*p*-Value	EA	AA	*p*-Value
Number of participants	31	11	20	-	16	15	-
Sex (male/female)	17/14	9/3	8/11	-	(10/6)	(7/8)	-
Age (years)	8.56 ± 1.34	8.21 ± 0.42	8.75 ± 0.29	-	8.12 ± 0.339	9.03 ± 0.311	-
Height (cm)	134.35 ± 12.39	130.38 ± 3.55	136.53 ± 2.78	0.190	130.53 ± 2.50	138.43 ± 3.53	0.076
Weight (kg)	36.04 ± 12.63	27.16 ± 1.89	40.33 ± 12.72	**0.002**	32.48 ± 2.49	39.92 ± 3.68	0.098
BMI (kg/m^2^)	19.42 ± 3.57	15.80 ± 0.40	21.41 ± 0.61	**0.000**	18.06 ± 0.82	20.29 ± 0.97	0.194
BMI z-score	1.27 ± 1.21	0.09 ± 0.31	2.03 ± 0.10	**0.000**	1.05 ± 0.32	1.50 ± 0.28	0.312

Note. Bold values are statistically significant at (*p* ≤ 0.05).

**Table 2:** A cluster of target IDs that were significantly differentially methylated between normal weight (NW) and overweight (OW)/obese (OB) children.

No.	Gene	No. of Significant Target IDs
1	*PCDHGA4*	52
2	*BRD2*	18
3	*PTPRN2*	15
4	*DIP2C*	13
5	*TAPBP*	13
6	*TGIF1*	13
7	*PITX2*	12
8	*COL9A3*	12
9	*PAX6*	11
10	*FDFT1*	11
11	*NKX6-2*	11
12	*SMC4*	11
13	*PCDHB16*	11
14	*GFI1*	10
15	*TRIM27*	10
16	*BAHCC1*	10
17	*HS3ST3B1*	10
18	*KIAA1949*	10
19	*MEIS2*	10
20	*NFIX*	10

**Table 3:** Cluster of target IDs that were significantly differentially methylated between European American (EA) and African American (AA) children.

No.	Gene	No. of Significant Target IDs
1	*PTPRN2*	10
2	*TNXB*	9
3	*MDLL1*	8
4	*PRDMI6*	7
5	*COC23A1*	6
6	*CSMD1*	6
7	*TRIM31*	6
8	*TTC7B*	6
9	*BAT5*	5
10	*EFR3B*	5
11	*LIMCH1*	5
12	*LRRC14B*	5
13	*NRG2*	5
14	*PALLD*	5
15	*RNF39*	5
16	*RPH3AL*	5
17	*RPS6KA2*	5
18	*WDR90*	5
19	*LHX5*	5
20	*PM20D1*	5

**Table 4:** GO (Gene Ontology) processes that were enriched due to DMRs between NW and OW/OB children.

Process	*p*-Value	Overlapping Genes
Carbohydrate kinase activity	0.000059	*FGGY*, *POMK*, *GALK2*, *HK2*, *GALK1*, *GNE*, *HK1*
Sequence-specific double-stranded DNA binding	0.000388	*PRDM4, RAX, RORC, HNRNPU, ZBTB20, ETS2, ELK3, NR3C2, ELK4, BASP1, CREB3L2, PITX2, SOX6, HES7, KCNH2, NKX2-8, NKX6-2, ZNF281, RFX3, SOX11, SOX12, HIC1, DMTF1, ZFP41, RFX7, RFX4, DDIT3, ATF3, ATF4, DLX2, DHX9, FOXG1, GATA5, BHLHE22, GLIS1, NHLH2, ALX3, ALX4, ZNF787, ZBED4, CPSF4, SMAD4, EGR3, ZNF32, NR1D2, SMARCA2, POU2F3, NR2F6, NFKB2, TFCP2, MAFF, MYF6, LHX6, LHX4, LHX9, TP73, MESP2, MCM8, HMGB2, BRCA1, CHD3, HOXC12, MEOX2, HOXC11, GLI2, ZIC1, KLF10, TGIF1, EMX1, PROP1, ARNT, PAX6, ZNF75A, SREBF2, KLF16, ETV7, SFPQ, IRF4, RARA, MCM4, PPARG, IRF9, HDAC4, YAP1, CEBPA, SRF, CEBPG, FOXK2, FOXK1, ZBTB45, ASCL1, ASCL2, HOXD10, NR2C1, GBX2, HSF1, TBX20, HSF4, E2F7, ZNF580*
GTPase regulator activity	0.002233	*ARHGAP11A, NRP1, SEC23A, LRRK2, ASAP3, GPS2, ASAP1, RASGRP3, SYDE2, PREX1, RIMS1, FLCN, ARHGAP20, RACGAP1, TBC1D14, SH3BP4, RGS20, BNIP2, SLIT2, TBC1D22A, TBC1D22B, RGS7, VAV3, GRTP1, ELMOD2, TNK2, TBCD, ELMOD3, ARHGAP29, ARHGAP26, ARFGAP3, RUNDC3A, ARHGAP10, EIF5, ARL2BP, GNAQ, SGSM2, PREB, RGS12, DEPDC5, PLCB1, SOS1*
Tubulin binding	0.002948	*GPAA1, LRRK2, DIXDC1, KIF14, FMN1, BRCA1, MAP1LC3B, APC2, AGTPBP1, MAP1S, SGIP1, DLEC1, KIF23, EML1, KIF6, CKAP5, KIF27, DNM2, EML5, EML6, KIF9, FAM161A, KIFC1, EFHC1, MAPRE3, ARHGEF2, ALDOA, ARL8A, TUBGCP2, NDRG1, GABARAP, SKA2, IFT74, RACGAP1, BLOC1S2, MAP6, WHAMM, MARK4, MAP9, MX1, TBCD, INO80, RAB11A, KIF18B, CDK5, REEP4, KIF26B, CCDC88C, TUBGCP5, DRG1, TUBGCP6, TRIM36*
γ-Tubulin binding	0.003088	*RACGAP1, TUBGCP2, BLOC1S2, DIXDC1, TUBGCP5, TUBGCP6, MARK4, NDRG1*

**Table 5:** GO (Gene Ontology) processes that were enriched due to DMRs between EA and AA children.

Process	*p*-Value	Overlapping Genes
Aspartic-type endopeptidase inhibitor activity	0.000059	*CRB2, ROCK1, NLRP7, BIN1*
Leucine zipper domain binding	0.004963	*PAWR, ERC1, PMF1*
Sterol binding	0.006447	*GRAMD1B, CD81, ERLIN1, TSPO, APOA1, OSBPL11, OSBPL10*
GTPase activator activity	0.007115	*RANBP2, ARHGEF10, RAB3IP, ELMOD1, ARHGEF10L, RASGRP2, ARHGAP26, ADAP1, RASGRP1, ARHGAP22, ABR, WNT11, NF1, SH3BP5, BNIP2, PLCB1, SERGEF, SOS1, TBC1D22A, RGL2, SBF2, DOCK1*
Cholesterol binding	0.009942	*GRAMD1B, CD81, ERLIN1, TSPO, APOA1, OSBPL10*

**Table 6:** KEGG (Kyoto Encyclopedia of Genes and Genomes) pathways that were enriched due to DMRs between NW and OW/OB children.

Pathway	*p*-Value	Overlapping Genes
Hippo signaling pathway	0.000429	*YAP1, GSK3B, LIMD1, PRKCZ, ACTB, ACTG1, SAV1, GLI2, PARD6B, PARD6A, CDH1, MYC, DVL3, WNT2, WNT4, TEAD3, APC2, WNT10A, SMAD4, WNT5B, FBXW11, WNT3A, WNT7B, WNT5A, BMP8A, FZD8, AXIN2, GDF6, PPP1CA, BMP2, PPP2R2B, DLG5, DCHS1, TP73*
Cushing syndrome	0.000750	*GSK3B, FH, ITPR3, CACNA1C, ADCY6, GNAI1, RAP1B, CACNA1I, PDE11A, RASD1, CREB3L2, DVL3, PDE8A, WNT2, WNT4, APC2, WNT10A, WNT5B, WNT3A, CDKN2A, WNT7B, WNT5A, FZD8, ARNT, AXIN2, NCEH1, GNAQ, AGTR1, GNAS, PLCB1, KCNK3, ATF4*
Folate biosynthesis	0.001785	*MOCS2, SPR, GCH1, FPGS, AKR1C3, ALPL, MOCS1, GPHN, PTS*
Hepatocellular carcinoma	0.002954	*GSK3B, SMARCD3, PTEN, TGFA, PIK3R2, ACTB, ACTG1, TERT, MYC, DVL3, PLCG1, WNT2, WNT4, APC2, WNT10A, SMAD4, WNT5B, GADD45B, WNT3A, BAD, CDKN2A, TXNRD1, WNT7B, WNT5A, ACTL6A, FZD8, AXIN2, SMARCA2, ARID1B, MTOR, PIK3CA, SOS1*
Proteoglycans in cancer	0.003846	*CD63, ROCK1, ROCK2, SDC2, IHH, TWIST1, PIK3R2, ITPR3, HOXD10, ACTB, ACTG1, MYC, FLNB, PLCG1, WNT2, WNT4, VAV3, WNT10A, WNT5B, WNT3A, WNT7B, MMP2, WNT5A, FZD8, ANK2, ANK3, ANK1, PTK2, MTOR, VAV2, PPP1CA, MAPK13, MRAS, PIK3CA, PDCD4, FAS, SOS1*
Basal cell carcinoma	0.004608	*APC2, GSK3B, WNT10A, WNT5B, GADD45B, WNT3A, WNT7B, WNT5A, FZD8, AXIN2, GLI2, BMP2, DVL3, WNT2, WNT4*
Type II diabetes mellitus	0.004951	*SOCS2, SOCS1, PKM, PIK3CA, PDX1, PIK3R2, CACNA1C, PRKCZ, CACNA1E, HK2, MTOR, HK1*
MAPK signaling pathway	0.008373	*RASGRF2, SRF, TGFA, CACNA1C, ECSIT, EFNA5, CACNA1E, DUSP16, FGF3, RASGRP3, ELK4, RPS6KA4, RAP1B, CACNA1I, PPP3R1, MYC, PDGFD, GNA12, FLNB, CD14, MAP3K6, DUSP4, NTRK1, DUSP3, GADD45B, CACNA2D1, PLA2G4A, HSPA2, IRAK4, MAPK8IP3, MAPK8IP1, CDC25B, NFKB2, TNFRSF1A, MAPK13, EFNA1, KITLG, MRAS, CACNB4, MAPKAPK3, DDIT3, FGF19, TAOK2, FAS, SOS1, CRK, PTPN5, ATF4*

**Table 7:** KEGG (Kyoto Encyclopedia of Genes and Genomes) pathways that were enriched due to DMRs between EA and AA children.

Pathway	*p*-Value	Overlapping Genes
Focal adhesion	0.000175	*SHC4, TNXB, FLT1, ITGB5, ROCK1, LAMC3, ITGB3, PDGFB, TNC, VEGFC, ACTN4, RELN, ITGA8, PIP5K1B, ITGB7, FYN, SOS1, DOCK1, PAK4*
ECM–receptor interaction	0.000391	*TNXB, RELN, SV2C, ITGB5, SV2B, LAMC3, ITGB3, TNC, ITGA8, ITGB7, HMMR*
Th1 and Th2 cell differentiation	0.006920	*IKBKB, NOTCH3, HLA-DRB5, STAT4, NFATC1, RUNX3, HLA-DRB1, HLA-DPA1, NFKBIB*
Arrhythmogenic right ventricular cardiomyopathy	0.007460	*CACNG8, ITGB5, ITGB3, TCF7, ITGA8, ITGB7, CACNA1C, CTNNA2*
Ras signaling pathway	0.012673	*SHC4, FLT1, PDGFB, VEGFC, KSR2, RASGRP2, RASGRP1, IKBKB, RASA3, GNG4, RASSF5, GNB1, NF1, SOS1, RGL2, PAK4*
Biosynthesis of unsaturated fatty acids	0.016499	*ELOVL1, ACOT7, ELOVL6, ACOX3*
Pathways in cancer	0.008461	*PTGER4, NOTCH3, ROCK1, PTGER1, LAMC3, NOTCH4, TCF7, PDGFB, ADCY8, RASGRP2, RASGRP1, IKBKB, RXRA, WNT11, MECOM, GNG4, RASSF5, STAT4, CTNNA2, SKP2, DAPK1, FZD6, VEGFC, AXIN2, DDB2, RUNX1, MSH3, G* *NB1, PLCB1, IL6ST, SOS1*

## Data Availability

The datasets used in the current manuscript are available from the corresponding author upon request.

## Supplementary Materials

The following supporting information can be downloaded at: https://www.mdpi.com/article/10.3390/genes14051098/s1, Table S1: DMRs between NW and OW/OB children; Table S2: Obesity related DMRs between NW and OW/OB children; Table S3: Non obesity-related target IDs between NW and OW/OB children; Table S4: Multinominal hierarchical regression analysis between obesity-related target IDs and BMI z-scores of children adjusted for race, maternal education, and family income; Table S5: DMRs between EA and AA children; Table S6: Obesity-related DMRs between EA and AA children; Table S7: Non-obesity-related DMRs between NW and OW/OB children.
